# Land use and cover maps for Mato Grosso State in Brazil from 2001 to 2017

**DOI:** 10.1038/s41597-020-0371-4

**Published:** 2020-01-27

**Authors:** Rolf Simoes, Michelle C. A. Picoli, Gilberto Camara, Adeline Maciel, Lorena Santos, Pedro R. Andrade, Alber Sánchez, Karine Ferreira, Alexandre Carvalho

**Affiliations:** 1 Brazil’s National Institute for Space Research (INPE), São José dos Campos, Brazil; 2 Group on Earth Observations (GEO), Geneva, Switzerland; 3 Institute of Applied Economic Research (IPEA), Brasília, Brazil

**Keywords:** Environmental sciences, Agriculture, Forestry, Developing world, Environmental impact

## Abstract

This paper presents a dataset of yearly land use and land cover classification maps for Mato Grosso State, Brazil, from 2001 to 2017. Mato Grosso is one of the world’s fast moving agricultural frontiers. To ensure multi-year compatibility, the work uses MODIS sensor analysis-ready products and an innovative method that applies machine learning techniques to classify satellite image time series. The maps provide information about crop and pasture expansion over natural vegetation, as well as spatially explicit estimates of increases in agricultural productivity and trade-offs between crop and pasture expansion. Therefore, the dataset provides new and relevant information to understand the impact of environmental policies on the expansion of tropical agriculture in Brazil. Using such results, researchers can make informed assessments of the interplay between production and protection within Amazon, Cerrado, and Pantanal biomes.

## Abstract (summary)

**Table:** 

Measurement(s)	land • land use
Technology Type(s)	computational modeling technique
Factor Type(s)	year • geographic location • land use and cover class
Sample Characteristic - Environment	land
Sample Characteristic - Location	Mato Grosso State

Machine-accessible metadata file describing the reported data: 10.6084/m9.figshare.11440461

## Background & Summary

Brazil is one of the top agricultural producers and exporters, being the largest extent of tropical rainforest and home to an estimated 15% to 20% of the world’s biodiversity. Such unique position leads to the need for balancing agricultural production and environmental protection^1^. Without substantial investments in productivity and strong land policies, the expansion of agricultural production in Brazil can be a significant factor in environmental degradation. It is vital to understand the impact of environmental policies on the expansion of tropical agriculture in Brazil.

In Nationally Determined Contribution (NDC) to the United Nations Framework Convention on Climate Change (UNFCCC) under the 2015 Paris Agreement, Brazil aims for zero illegal deforestation and zero net emissions within the Amazon rainforest by 2030. The forest emission balance will be achieved by restoring and reforesting 12 million hectares. Brazil’s NDC also makes a firm commitment to promote low-carbon agriculture and to increase biofuel use for transportation. Overall, achieving the emission reduction goals Brazil set in its NDC will highly depend on how the country meets the targets associated with the land use sector.

Since the election of the current Brazilian president in late 2018, there is a growing tension between the interests of Brazilian agricultural exporters and rural producers mostly linked to extensive cattle ranching. While the export sector supports the country’s pledges to the Paris Agreement, most cattle ranchers and smallholders do not want to commit to environmental protection policies^2^. Since the traditional rural sector is one of the primary supporters of current government, there are increasing concerns about whether Brazil will be committed to achieve its NDC. Comparing the environmental impact of different agricultural sectors is therefore important for all those interested in land policies in Brazil.

The legal basis for land policies in Brazil is the Forest Code. When created in 1965, it established a proportion of rural properties that must be permanently maintained as forest (legal reserve). It also prohibited clearing vegetation in sensitive areas such as steep slopes and along riverbanks and streams. In 2012, Congress approved a revision of the Forest Code. It stipulates that landowners, in the Legal Amazon, must conserve 80% of their property in forest areas, 35% in cerrado areas, 20% in general fields. To monitor compliance with the new Forest Code, Brazil has been successfully using wall-to-wall satellite-based monitoring^3^. Using satellite observations allows consistent monitoring of land use change, which is necessary for assessing how effective have been the enforcement of public policies as well as the new Forest Code^4^.

**Fig. 1:**
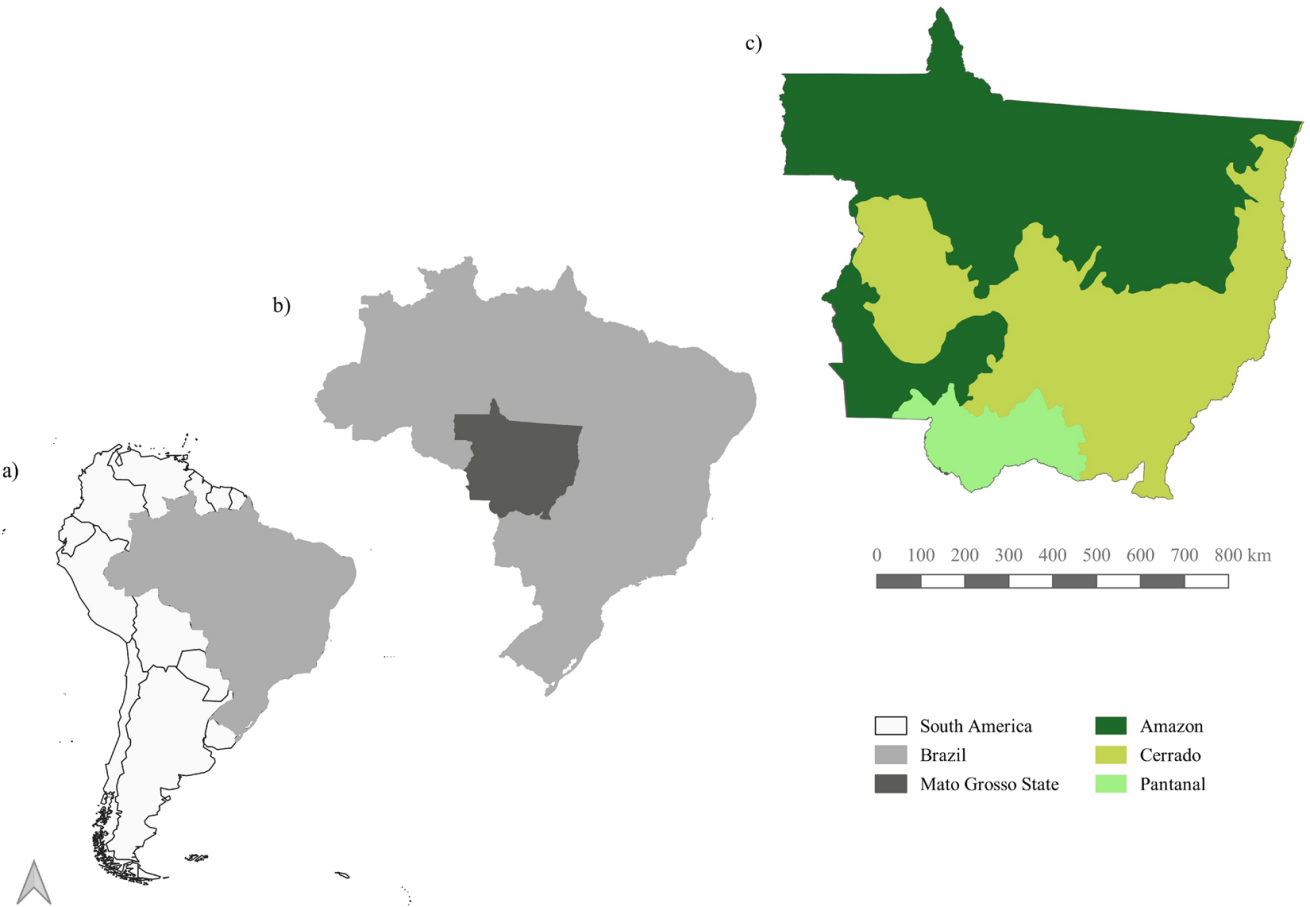
Location and characterization of the study area. (**a**) Brazil relative to South America continent; (**b**) Mato Grosso State relative to Brazil; (**c**) Mato Grosso State biomes.

One particular area of interest for understanding the balance between production and protection in Brazil is the Mato Grosso State, one of the world’s most extensive agricultural frontiers^5–8^. Mato Grosso is the third largest state of Brazil, with an area of 90,335,700 ha. If it was a country, it would be the world’s 33rd largest one, being almost as large as Venezuela and Nigeria. Mato Grosso also contains part of three Brazilian biomes: Amazon, Cerrado, and Pantanal (Fig. 1). From 1988 to 2018, Brazil’s National Institute for Space Research (INPE) estimates that 14.5 million ha of natural forests in the Amazonia biome in Mato Grosso have been clear-cut. INPE also estimates that, from 2001 to 2018 in Mato Grosso, more 4.5 million ha of natural cerrado vegetation have been removed.

Based on the above motivation, this paper describes a dataset of yearly land use and land cover maps for Mato Grosso from 2001 to 2017. These maps are temporally consistent and provide information on deforestation and changes in natural vegetation, crop and pasture expansion, a well as productivity increase. To ensure multi-year compatibility, the work uses Moderated Resolution Imaging Spectroradiometer (MODIS) sensor analysis-ready products and an innovative method that applies machine learning techniques to classify satellite image time series. Using the results, it is possible to make informed assessments of the interplay between production and protection in the Amazon, Cerrado, and Pantanal biomes.

## Methods

**Fig. 2:**
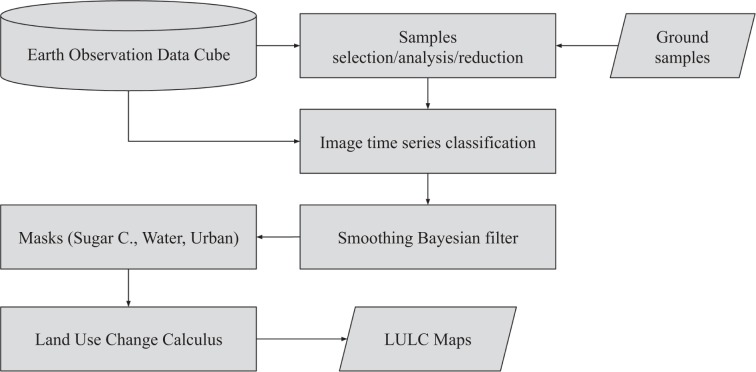
Diagram depicting our methods.

In this section, we detail our approach to generate land use and cover maps of Mato Grosso State. The main steps are depicted in Fig. 2.

### Input Data

We based our work on Earth observation data cube, using data stored in a cloud service (Amazon Web Services), over which we ran the classification. The input data is a set of MOD13Q1 collection 6 images, provided by NASA/LPDAAC from 2000-09-01 to 2017-08-31, covering Mato Grosso State. The MOD13Q1 images are available every 16 days at a 250-meter spatial resolution in the sinusoidal projection^9^. For our analysis, we used normalized difference vegetation index (NDVI), enhanced vegetation index (EVI), near-infrared (NIR), and mid-infrared (MIR) attributes.

The samples dataset has 2,115 samples containing longitude, latitude, start date, end date, and label. We defined nine land use and cover classes: (1) forest, (2) cerrado, (3) pasture, (4) soy-fallow (single crop), (5) fallow-cotton (single crop), (6) soy-cotton (double crop), (7) soy-corn (double crop), (8) soy-millet (double crop), and (9) soy-sunflower (double crop). Our samples range from 2000 to 2015 and all samples are available at PANGAEA repository^10^. The crop and pasture ground data was collected through field observations and farmer interviews provided by^7,11^. Samples for cerrado and forest were provided through fieldwork and high-resolution images. Ground samples for soybean-fallow were provided through fieldwork, based on previous work of^5^. The classes are shown in Table 1.

**Table 1:** Samples used for training the classification model.

Class label	Count	Frequency
Cerrado	379	20.0%
Fallow-Cotton	29	1.5%
Forest	131	6.9%
Pasture	344	18.2%
Soy-Corn	364	19.2%
Soy-Cotton	352	18.6%
Soy-Fallow	87	4.6%
Soy-Millet	180	9.5%
Soy-Sunflower	26	1.4%

We retrieved time series data of the 2,115 samples from the Web Time Series Service (WTSS)^12^, an R package available on CRAN (https://CRAN.R-project.org/package=wtss). Each sample corresponds to one year of observations that comprises 23 values of MOD13Q1 per band.

**Fig. 3:**
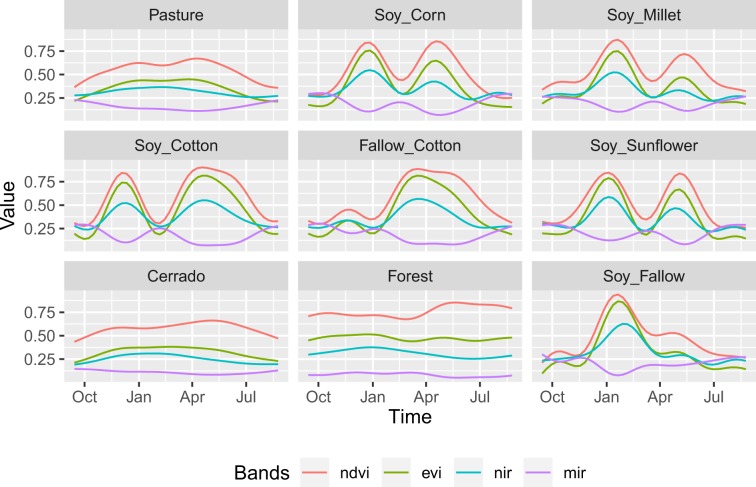
Temporal patterns of NDVI, EVI, NIR, and MIR bands for the land use and cover classes. The patterns are obtained using a generalized additive model on time series of samples dataset. Source^8^.

The temporal patterns of the ground samples (Table 1), using NDVI, EVI, NIR, and MIR bands, can be seen in Fig. 3, which uses a generalized additive model to estimate the joint distribution of the samples dataset for each class^13^.

### Pre-processing for sample quality control

One of the key challenges when using samples to train machine learning classification models is assessing their quality. Noisy and imperfect training samples can have a negative effect on classification performance^14^. Therefore, it is useful to apply pre-processing methods to improve the quality of the samples and to remove those that might have been wrongly labelled or that have low discriminatory power. In this work, we applied a clustering method based on self-organizing maps (SOM) neural network to test sample quality.

Self-organizing maps is a dimensionality reduction technique^15^. High-dimensional data is mapped into two dimensions, keeping the topological relations between data patterns. This allows users to visualize and assess the structure of the input dataset. For quality control of the training data, we used a SOM clustering method, whose input is a time series samples dataset. The output layer comprises a 2D grid of neurons, each associated to a weight vector of the same dimension as the input space.

In the SOM algorithm, the 2D grid of neurons is initialized randomly. Then, for each time series sample, the algorithm finds the neuron with the smallest distance to the sample, based on its weight vector. After the match, the neuron’s weight vector and those of its neighbors are then updated. After all training samples are associated with neurons, each neuron is labelled using a majority vote, taking the most frequent class from the samples associated with it. In this way, SOM splits the output space into regions. It is expected that good quality samples of each class would be close together in the resulting map.

To increase the reliability of quality control procedures, SOM was executed several times. Using this iterative procedure, we computed the probability of each sample belonging to the resulting clusters. From these probabilities, we analyzed the separability of samples with similar phenological patterns and decided which samples were to be discarded. This allows using SOM to detect and remove outliers. Table 2 illustrates how this method works. It contains three samples of pasture, identified from 1 to 3. For sample 1, its original and clustered label matched 100% of the time. For sample 2, only 52% of the original and cluster labels matched. Finally, sample 3 matched only 5% of the original label. We define good samples as those that match at least 80% of the time. Thus, in this example, samples 2 and 3 were removed from the data set.

**Table 2:** Reliability of three pasture samples, identified as 1, 2, and 3.

Identifier	Original class	Cluster label	Frequency
1	Pasture	Pasture	100%
2	Pasture	Pasture	52%
		Cerrado	41%
		Forest	2%
		Soy-Corn	2%
		Soy-Cotton	2%
		Fallow-Cotton	1%
3	Pasture	Cerrado	94%
		Pasture	5%
		Forest	1%

For Mato Grosso samples, the SOM-based clustering reduced the training dataset by 10.5%, from 2,115 to 1,892 entries. This filtered dataset was then used to train the classification model.

### Image time series classification

To generate our maps, we used support vector machine (SVM) as a classification model. Given a multidimensional dataset, SVM finds an optimal separation hyperplane that minimizes misclassifications^16^. For data that is not linearly separable, SVM includes kernel functions that map the original feature space into a higher dimensional space, providing nonlinear boundaries to the original feature space. SVM is one of the most widely used algorithms in machine learning applications and has been widely applied to classify remote sensing data^17^.

In a recent review of machine learning methods to classify remote sensing data^18^, the authors note that many factors influence the performance of these classifiers, including the size and quality of the training dataset, the dimension of the feature space, and the choice of the parameters. With Mato Grosso data, previous experiments by the authors have shown that the SVM classifier has a better performance than other machine learning methods such as random forest for MOD13Q1 time series data^8^.

For SVM training, we used a 92-dimensional feature space, comprising four time series for each pixel. Each time series contains 23 samples of one of the MOD13Q1 bands NIR, MIR, EVI, and NDVI. The dataset of 1,892 labelled and quality-controlled time series was used to train an SVM model using a radial basis kernel function (RBF), with cost $C=1$ and *γ* = 1/92^19^. We chose these parameters based on a 5-fold cross-validation test. Parameter *C* is the cost used as a softened parameter of hyperplane boundaries, while *γ* is a distance normalization parameter used in the RBF kernel. We used sits R package to train SVM and to classify all MOD13Q1 tiles of Mato Grosso stored in an AWS S3 service.

### Local smoothing by Bayesian filtering

One of the well-established methods in remote sensing image analysis is to combine pixel-based classification methods with a spatial post-processing method to remove outliers and misclassified pixels. Methods proposed in the literature include modal filters^20^ and probabilistic relaxation^21^. Our method uses Bayesian smoothing to reclassify the pixels.

Usually, machine learning methods assign class probabilities to each pixel. Most applications using this approach select the most probable class from the classifier output to be the categorical result for each pixel. The proposed method uses all pixel classes’s probabilities to compute the resulting confidence. When the magnitude of the discrepancies among the pixel probabilities is high, we have a higher confidence in the classification. Otherwise, when the probabilities have similar magnitudes, we have a low confidence. This is a typical situation in borders and mixed pixels.

To change low confidence pixels, we followed well-established Bayesian smoothing methods^22^: borrow strength from the neighbors to reduce the variance of the estimated class for each pixel. The main rationale is to use Bayesian inference considering the mean and variance of the pixel’s neighbors to calculate the posterior Bayesian probabilities, then reevaluate the most probable class for the pixel. This procedure can change the class of pixels with low confidence to the neighborhood class with a higher confidence. When the local class variance of the neighbors is high, the method gives more weight to the original pixel value. The smoothing algorithm considers a global parameter ${\sigma }^{2}$ that weighs the smoothness level by increasing the influence of the neighborhood. When ${\sigma }^{2}=0$, there is no change in the posterior Bayesian class probabilities. Positive values of ${\sigma }^{2}$ indicate the influence the neighbors in the posterior Bayesian probabilities.

In our case, after some classification tests, we set ${\sigma }^{2}=10$ for all classes, which showed the best performance in the technical validation. Additionally, we used a single neighborhood rule, being all those pixels with Chebyshev distance equal to one, which is the same as to consider a 3 × 3 window around a pixel.

### Post-processing: masks and land use change calculus

Three land cover classes that are not included in the training dataset were introduced as masks on all output maps: sugarcane, water, and urban areas. The sugarcane mask from 2003 to 2016 comes from Canasat project^23^, which maps sugarcane areas in the South-Central region of Brazil using LANDSAT images^24^. The water mask comes from^25^, who used three million LANDSAT satellite images to quantify changes in global surface water over the past 32 years (1984 to 2015). Finally, the urban area mask was provided by^26^.

To prepare the base map (year 2001), we considered using the Deforestation Monitoring Project (PRODES) Amazon (http://www.obt.inpe.br/prodes/) and PRODES Cerrado (http://www.obt.inpe.br/cerrado/) datasets produced by Brazil’s National Institute for Space Research (INPE). We used PRODES datasets to achieve a better consistency in our base map, as our classification method is unaware of preceding land cover trajectories before 2001. These datasets are the official Brazilian statistics on deforestation^27^. Table 3 lists the set of rules applied on the base map using these datasets. We applied each rule comparing corresponding pixels of two maps. We denote ${{\rm{M}}}_{i}$, ${{\rm{A}}}_{i}$, and ${{\rm{C}}}_{i}$ as the classes of pixel $i$ of Mato Grosso classification, PRODES Amazon, and PRODES Cerrado maps, respectively. The result of each rule is a new class for ${{\rm{M}}}_{i}$, described in the right column.

**Table 3:** Rules applied on the base map (year 2001).

Condition	Resulting class for M_*i*_
${{\rm{A}}}_{i}=\langle Forest\rangle$	$\langle Forest\rangle$
${{\rm{A}}}_{i}=\langle Non$-$Forest\rangle$ and ${{\rm{M}}}_{i}=\langle Forest\rangle$	$\langle Cerrado\rangle$
${{\rm{A}}}_{i}=\langle Deforestation\rangle$ and ${{\rm{M}}}_{i}=\langle Forest\rangle$	$\langle Sec$-$Vegetation\rangle$
${{\rm{C}}}_{i}=\langle Non$-$Anthropized\rangle$ and ${{\rm{M}}}_{i}=\langle Forest\rangle$	$\langle Cerrado\rangle$
${{\rm{C}}}_{i}=\langle Anthropized\rangle$ and ${{\rm{M}}}_{i}=\langle Forest\rangle$	$\langle Sec$-$Vegetation\rangle$

Since our method produces maps independently, the results may have temporal inconsistencies. For example, a natural forest area that was cut in one year may regrow back to forest after being abandoned. In this case, it is useful to distinguish pristine forest from secondary vegetation, which is also classified as a forest in later years. Another example is the case when a pixel classified as forest in one year is classified as cerrado in another year, which represents an impossible transition. To handle these inconsistencies, we used the Land Use Change Calculus (LUC Calculus)^28^ over all produced maps as a set of land use trajectories from 2001 to 2017, using 2001 as a reference date.

Table 4 expresses the set of rules that describe when a particular class will be replaced by another, using LUC Calculus. We considered four classes: forest (F), cerrado (C), pasture (P), and soybean (S) (any class with soybean). The rules were applied sequentially to ensure the temporal consistency among classes over the years. The expressions indicate that trajectories on the left side of ‘→’ are updated to the classes on the right side. The updated classes are those highlighted with ‘*’ symbol. All rules assume 2001 as the base map. Rules nine and ten create a new class for secondary vegetation (SV). This class represents deforested areas that regrew as a secondary forest after being abandoned.

**Table 4:** Rules applied over all classified years.

Land-use transition rules	
1. C → F*	$\Rightarrow$ C → C*
2. C → C → P* → C	$\Rightarrow$ C → C → C* → C
3. C → C → S* → C	$\Rightarrow$ C → C → C* → C
4. P → P → C* → C* → P	$\Rightarrow$ P → P → P* → P* → P
5. F → C* → F → F	$\Rightarrow$ F → F* → F → F
6. F → F → C* → F	$\Rightarrow$ F → F → F* → F
7. F → C* → F	$\Rightarrow$ F → F* → F
8. F → C*	$\Rightarrow$ F → F*
9. F → F → P → F*	$\Rightarrow$ F → F → P → SV*
10. P → P → F* → P	$\Rightarrow$ P → P → SV* → P

The classes labels: forest (F), cerrado (C), pasture (P), soybean (S), and secondary vegetation (SV).

**Fig. 4:**
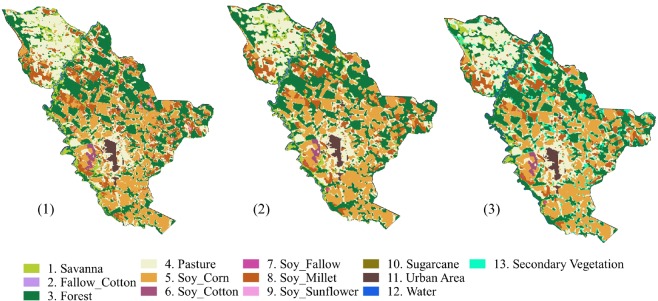
SVM classification for Sinop municipality in 2016. (1) Original map with masks; (2) original map with Bayesian smoothing and masks; (3) final map after applying the LUC Calculus and masks.

Figure 4 displays an example of how post-processing was applied. It shows different results for Sinop municipality in 2016. Figure 4-(1) illustrates the original SVM output map, after applying water, sugarcane, and urban masks. Figure 4-(2) shows the result after Bayesian smoothing on the original classification. Finally, Figure 4-(3) shows the output map after LUC Calculus. This last map is the only one that identifies secondary vegetation areas.

## Data Records

The dataset provides annual land use and cover maps from 2001 to 2017 in sinusoidal projection, which is the same cartographical projection used by the input MODIS images. The archive available at PANGAEA^10^ contains the classified maps in compressed TIFF format (one per year) at MODIS resolution, as well as a file with the training dataset (1,892 ground samples) in CSV format and a style file for displaying the data in QGIS.

## Technical Validation

Quality assessment using a 5-fold cross-validation^30^ of the training samples indicates an overall accuracy of 96%. Table 5 shows the user’s and producer’s accuracy for each land use and cover classes.

**Table 5:** Summary of k-fold cross-validation accuracy estimation.

Class	User acc.	Prod acc.
Cerrado	98%	99%
Fallow-Cotton	96%	93%
Forest	99%	98%
Pasture	97%	98%
Soy-Corn	91%	93%
Soy-Cotton	97%	97%
Soy-Fallow	98%	98%
Soy-Millet	90%	89%
Soy-Sunflower	77%	65%

The cropland area increased from 2001 to 2017. This trend is corroborated by^6,7,31^. A decreasing on soybean area between 2005 to 2007 was also observed by^6,32^. Moreover, the use of double-crop systems, involving soybeans in the first cycle and some other commercial crops in the second cycle, also increased from 2001 to 2017. This is in accordance with^7^.

**Fig. 5:**
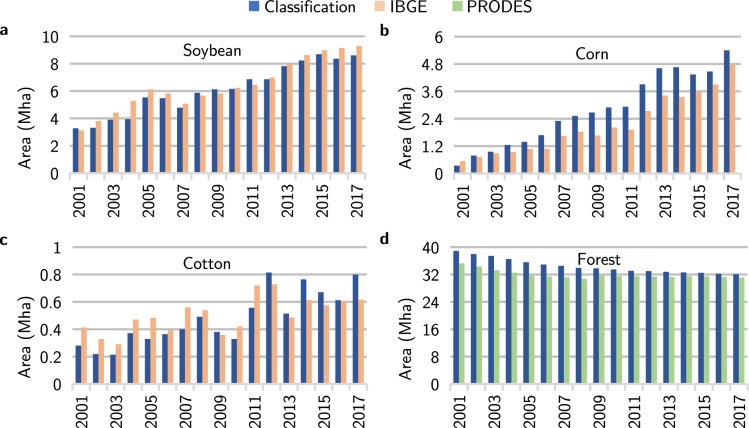
Comparison of land use areas from 2001 to 2017. Total area of (**a**) soybean, (**b**) corn, (**c**) cotton, and (**d**) forest in Mato Grosso estimated by the proposed classification method, together with IBGE cropland survey and PRODES.

The correlation coefficient between the agricultural areas classified by our method and the official crop statistics by the Brazilian Institute of Geography and Statistics (IBGE)^33^, for harvests from 2001 to 2017, was equal to 0.98. At the state level, soybean, cotton, corn, and sunflower areas had a correlation equal to 0.97, 0.85, 0.98, and 0.80 respectively, as shown in Fig. 5.

Compared to the IBGE statistics, the classification slightly underestimates soybean areas in most years. It also underestimates cotton areas until 2012 and overestimates corn areas. IBGE statistics are based on questionnaires and not on systematic surveys, which might produce inaccurate estimates. Additionally, these differences may have been caused by the spatial resolution of MODIS (250 meters), which generates spectral mixing for different land uses within a single pixel^34^.

Forest area has a correlation of 0.88 if compared with data of PRODES^35^. The classification method overestimated forest areas in Mato Grosso, possibly because some cerrado areas are very dense and spectrally similar to forest. We also compared the areas classified as forest with the Global Maps of 21st-Century Forest Cover Change produced by^36^, for the year 2000. We found that 98% of the pixels classified as forest match the pixels above 25% of tree cover.

**Fig. 6:**
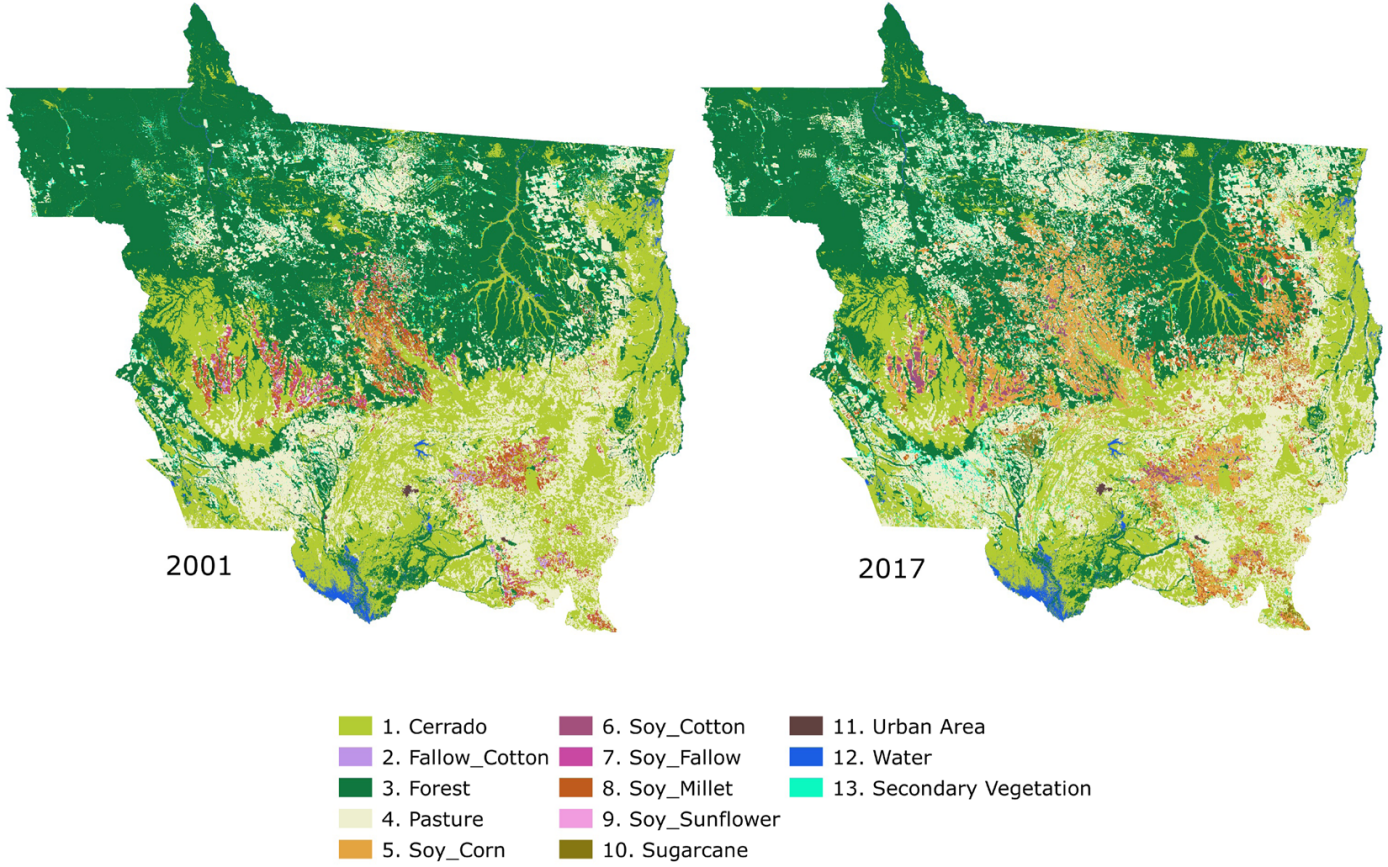
Classified maps of Mato Grosso in 2001 and 2017, with sugarcane, urban area and water masks.

Our results show an expansion of pasture area in the Mato Grosso State between 2001 and 2017. We can observe in Fig. 6 that pasture expansion occurred mainly in the north, within the Amazon biome. A similar finding is observed by^37^.

All these validation results show that our maps are consistent and reliable. However, the spatial resolution of MODIS images (250 m) have limitations to represent some areas as small crop fields, water bodies, and urban areas.

## Usage Notes

The land use and cover dataset contributes to understanding land use changes and trends. It provides essential information for public policy, decision making, environmental studies, territorial planning, and law and agreements enforcements. In this section, we provide two applications examples of land use and cover dataset.

The dataset can provide information to assist in the fulfillment of Brazilian NDC, which commit to reduce greenhouse gas emissions (GHG) by 37% below 2005 levels in 2025 and 43% until 2030. Actions comprising land use, renewable energy, and low carbon agriculture sectors compose key elements of the Brazilian commitments. Land use and cover maps are fundamental for monitoring land use change trends. These maps can also be used as input to complex models for GHG inventories to verify if the Brazilian NDC has been fulfilled for climate mitigation.

The demand for agricultural land is one of the main drivers of land use change in Brazil. There are already public political aggravations to slow down the expansion, especially of soybeans (whose Mato Grosso is the largest producer state in the world) and encourage the intensification of agriculture, as for example soybean moratorium (signed in 2006) and the new Forest Code (NFC) (signed in 2012). Another potential usage of the dataset is to track the agriculture intensification in Brazil, and on which land use agriculture has expanded. It is also possible to verify compliance with the soybean moratorium and the NFC policies.

## Data Availability

The code used in this study was provided under the GNU General Public Licence v3.0 and is available in^29^. The images were processed and classified on Amazon Web Services (AWS). The R package *sits* (Satellite Image Time Series) provides a set of visualization methods for an image time series, clustering methods for the time series samples, machine learning methods for the time series classification, including SVM, LDA, QDA, GLM, and other tools that support analysis of long-term satellite image time series. The development version of *sits* is available on GitHub at https://github.com/e-sensing/sits.
